# Study on the Factors Affecting the Self-Healing Performance of Graphene-Modified Asphalt Based on Molecular Dynamics Simulation

**DOI:** 10.3390/polym16172482

**Published:** 2024-08-30

**Authors:** Fei Guo, Xiaoyu Li, Ziran Wang, Yijun Chen, Jinchao Yue

**Affiliations:** 1 School of Water Conservancy and Transportation, Zhengzhou University, Zhengzhou 450001, China; 2 Henan Central Construction Engineering Co., Ltd., Zhengzhou 450016, China

**Keywords:** molecular dynamics, self-healing performance, oxidative aging, graphene-modified asphalt, healing temperature, damage degree

## Abstract

To comprehensively understand the impact of various environmental factors on the self-healing process of graphene-modified asphalt, this study employs molecular dynamics simulation methods to investigate the effects of aging degree (unaged, short-term aged, long-term aged), asphalt type (base asphalt, graphene-modified asphalt), healing temperature (20 °C, 25 °C, 30 °C), and damage degree (5 Å, 10 Å, 15 Å) on the self-healing performance of asphalt. The validity of the established asphalt molecular models was verified based on four physical quantities: density, radial distribution function analysis, glass transition temperature, and cohesive energy density. The simulated healing time for the asphalt crack model was set to 200 ps. The following conclusions were drawn based on the changes in density, mean square displacement, and diffusion coefficient during the simulated healing process under different influencing factors: Dehydrogenation and oxidation of asphalt molecules during the aging process hinder molecular migration within the asphalt crack model, resulting in poorer self-healing performance. As the service life increases, the decline in the healing performance of graphene-modified asphalt is slower than that of base asphalt, indicating that graphene-modified asphalt has stronger anti-aging properties. When the vacuum layer in the asphalt crack model is small, the changes in the diffusion coefficient are less pronounced. As the crack width increases, the influence of various factors on the diffusion coefficient of the asphalt crack model becomes more significant. When the crack width is large, the self-healing effect of asphalt is more dependent on these influencing factors. Damage degree and oxidative aging have a more significant impact on the healing ability of graphene-modified asphalt than healing temperature.

## 1. Introduction

As the service life of asphalt pavements increases, cracking and damage become more severe [1,2,3]. To develop asphalt concrete pavements with excellent road performance and long service life, many scholars have studied the impact of various external environmental factors and internal factors on the self-healing process of asphalt [4,5,6]. After decades of research, numerous experimental methods for studying the self-healing performance of asphalt have been developed [7,8]. These experimental methods can be divided into field tests and laboratory tests based on the testing environment. Field test methods assess the damage recovery of asphalt pavements by measuring the stiffness of the pavement before and after rest periods. Due to the complexity of field testing, most methods for evaluating asphalt self-healing performance are laboratory-based. Common laboratory tests include the three-point bending and four-point bending tests for asphalt mixtures [9,10], fatigue–healing–fatigue tests based on DSR [11], and intermittent loading fatigue tests [12]. However, the strength recovery observed in macroscopic mechanical performance tests (such as those based on fracture or fatigue) is not solely attributable to the self-healing properties of asphalt but may also result from viscoelastic responses (such as non-linearity, thixotropy, spatial hardening, self-heating, etc.) occurring during the unloading phase.

Traditional repair methods for asphalt pavements require manual intervention and can cause traffic disruptions and resource consumption [13,14]. Consequently, preventive maintenance techniques have gained significant attention from researchers. The core of preventive maintenance is to enhance the self-healing ability of asphalt materials to extend the service life of asphalt pavements [15,16]. The main theories regarding asphalt self-healing include the capillary flow theory, phase field theory, molecular diffusion theory, and surface energy theory. Fan et al. [17] established a healing model for asphalt mixtures based on the capillary flow theory and compared the performance of AC-13 and AC-20 mixtures, finding that AC-13 exhibited superior healing performance. Bhasin et al. [18] developed a preliminary phase field model to simulate the microstructural evolution of the four components of asphalt, explaining the damage and self-healing evolution of asphalt binders at the micron scale. Sun et al. [19] discovered, based on molecular diffusion theory, that when the temperature is above 273 K, the diffusion coefficient of SBS-modified asphalt is higher than that of pure asphalt, indicating a greater healing potential for SBS-modified asphalt. Zhang et al. [20] quantitatively evaluated the healing performance of DCLR-modified asphalt and its combination with typical aggregates based on the surface free energy theory. Xiao et al. [21] combined molecular diffusion theory with surface energy theory and proposed a new index, the healing factor, which comprehensively considers the diffusion characteristics and energy changes of asphalt molecules to evaluate self-healing performance. However, each theory has certain limitations and lacks the capability to comprehensively explore the effects of different influencing factors on the self-healing performance of asphalt. The capillary flow theory explains the self-healing mechanism of asphalt materials primarily under high-temperature conditions but lacks explanations for low-temperature behavior. The phase field theory, which uses atomic force microscopy to study microstructural changes during asphalt cracking and healing, does not fully and accurately describe the self-healing mechanism. Surface energy theory can theoretically analyze the self-healing process but requires extensive experimental validation. Molecular dynamics simulations, based on molecular diffusion theory, offer a new method to study the microscopic mechanisms of asphalt materials, providing insights into the healing process at the micro scale that cannot be observed through conventional experimental methods.

In the 1960s, dynamic simulations began to be applied to asphalt research [22]. Today, molecular dynamics simulations have been widely used in the study of various properties of asphalt materials. The application of molecular dynamics simulation methods allows for the investigation of asphalt self-healing performance. By establishing vacuum layers of different thicknesses between two asphalt molecules to simulate varying crack widths and controlling temperature, pressure, time, and asphalt molecule types during the simulation process, researchers can compare the self-healing performance of asphalt under different influencing factors. As a novel nanomaterial, graphene has gradually become a research hotspot in the field of modified asphalt due to its excellent mechanical and electrical properties. Li et al. [23] discovered that the high thermal conductivity of graphene accelerates the self-healing process of asphalt materials when heated. Liu et al. [24] observed, using SEM, that graphene forms a network structure in the asphalt matrix, enhancing its uniformity and stability. The microstructure of graphene-modified asphalt is denser, resulting in faster crack healing. Wang et al. [25] found that graphene-modified asphalt exhibits high self-healing capacity and extended fatigue life during multiple damage–repair cycles. Wang et al. [26] evaluated the long-term performance of graphene-modified asphalt, showing excellent self-healing properties and improved durability under various climatic conditions. Gong et al. [27] incorporated two types of carbon nanotubes and graphene into an average molecular model of asphalt to study the effect of modifiers on asphalt self-healing performance. The results showed that the addition of carbon nanotubes improved the self-healing performance of base asphalt, while the effect of graphene on the self-healing performance of base asphalt was not significant. Qu et al. [28] found that graphene-modified asphalt exhibited enhanced self-healing performance. The discrepancy between the two studies could be due to the different base asphalt molecular models selected in the simulations. Sun et al. [29] studied the self-healing mechanisms of asphalt molecules through molecular dynamics simulations and proposed three indicators to evaluate the self-healing ability of asphalt binders (diffusion coefficient, activation energy, and pre-exponential factor). Different factors have varying effects on the performance of materials [30]. However, there is currently no unified set of evaluation criteria for the impact of aging degree, asphalt type, healing temperature, and damage degree on the self-healing performance of asphalt in molecular dynamics simulations.

The self-healing process of asphalt involves the diffusion and miscibility of asphalt molecules. In the dynamics simulation, this is demonstrated by the movement of asphalt molecules along with the force field and their tendency to diffuse outward from their initial positions. The healing performance of asphalt is closely related to its composition and molecular structure, involving energy changes and molecular diffusion phenomena during the healing process [5,31,32]. This study focuses on base asphalt and graphene-modified asphalt with different aging degrees. By constructing healing models under various healing temperatures and crack widths, we simulate and analyze the self-healing behavior of asphalt under conditions of different aging degrees, asphalt types, healing temperatures, and crack widths. A four-component twelve-molecule system was used to establish an AAA-1 asphalt molecular model. The self-healing behavior of asphalt was simulated and analyzed under three aging degrees (unaged, short-term aged, and long-term aged), two types of asphalt (base asphalt and graphene-modified asphalt), three healing temperatures (20 °C, 25 °C, and 30 °C), and three crack widths (5 Å, 10 Å, and 15 Å). The self-healing performance of asphalt was evaluated using multiple indicators from both qualitative (density) and quantitative (diffusion coefficient) perspectives.

## 2. Establishment and Validation of Asphalt Molecular Models

### 2.1. Establishment of the Base Asphalt Model

**Figure 1:**
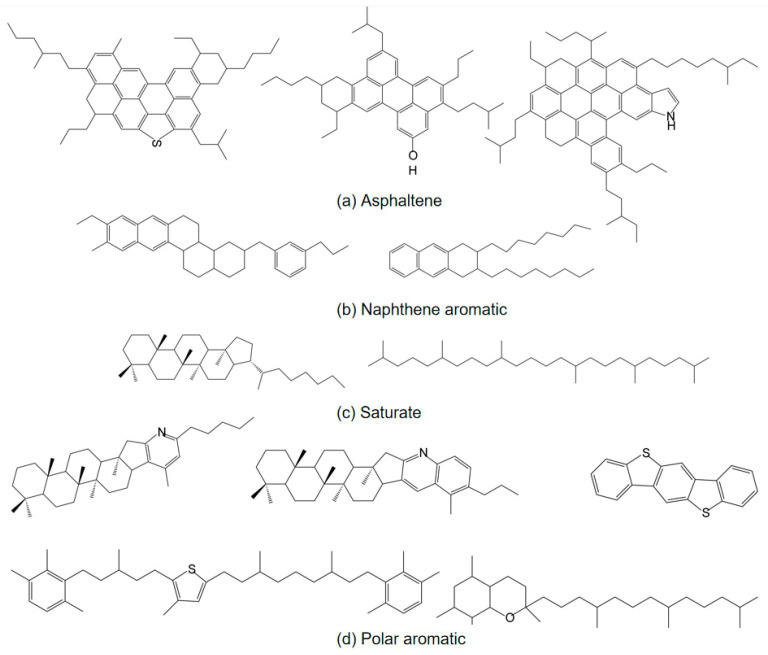
Asphalt molecular structure.

**Figure 2:**
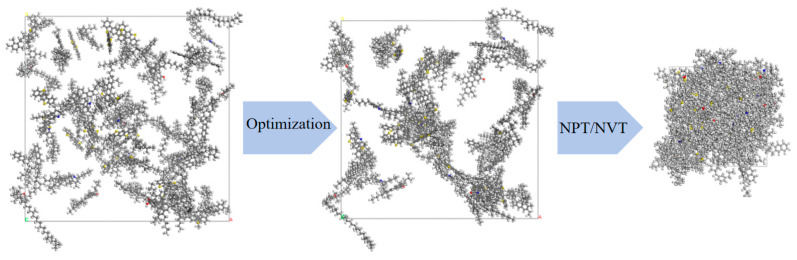
Asphalt molecular model (grey for carbon atoms, white for hydrogen atoms, yellow for sulfur atoms, red for oxygen atoms, and blue for nitrogen atoms).

The AAA-1 asphalt molecular model [33] was selected for this study. The modeling process is as follows:

(1) Using Materials Studio 2019 software, the molecular structures of twelve types of asphalt molecules were drawn, as shown in Figure 1. All the drawn molecular structures were imported into the Amorphous Cell module. According to the parameters in Table 1, the number of each type of molecule in a unit cell was input into the software. The initial density was set to 0.1 g/cm^3^, and the initial temperature was set to 295.15 K. Due to the presence of long-chain structures among the twelve types of asphalt molecules, setting a low initial density ensures ample space within the unit cell, keeping the molecules sufficiently dispersed to prevent entanglement, which could affect subsequent molecular dynamics simulations;
(2) The asphalt molecular model established in the previous step was subjected to geometry optimization and annealing, both performed within the COMPASSⅡ force field. During the geometry optimization process, the step number was set to 100,000. For the annealing process, the temperature ranged from 300 K to 500 K, with 10 cycles;
(3) After geometry optimization and annealing, the asphalt molecular model was subjected to a 1 ns dynamics calculation within the Canonical Ensemble (NVT) at a temperature of 298.15 K to obtain a uniform asphalt molecular model. Subsequently, a 1 ns dynamics calculation was performed within the Isothermal-Isobaric Ensemble (NPT) at a temperature of 298.15 K and a pressure of 1 standard atmosphere to compress the volume of the asphalt molecular model. The final molecular model is shown in Figure 2.

### 2.2. Establishment of Oxidized Aging Asphalt Model

**Figure 3:**
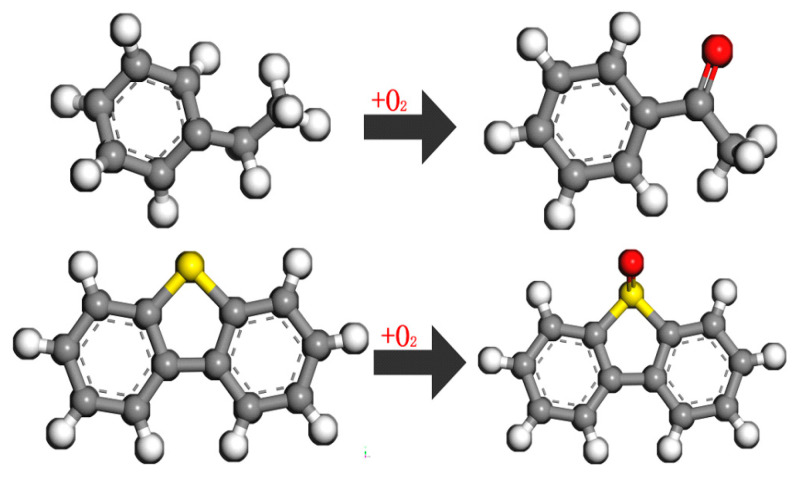
Oxygen-containing functional group model (gray for carbon atoms, white for hydrogen atoms, yellow for sulfur atoms, and red for oxygen atoms).

**Figure 4:**
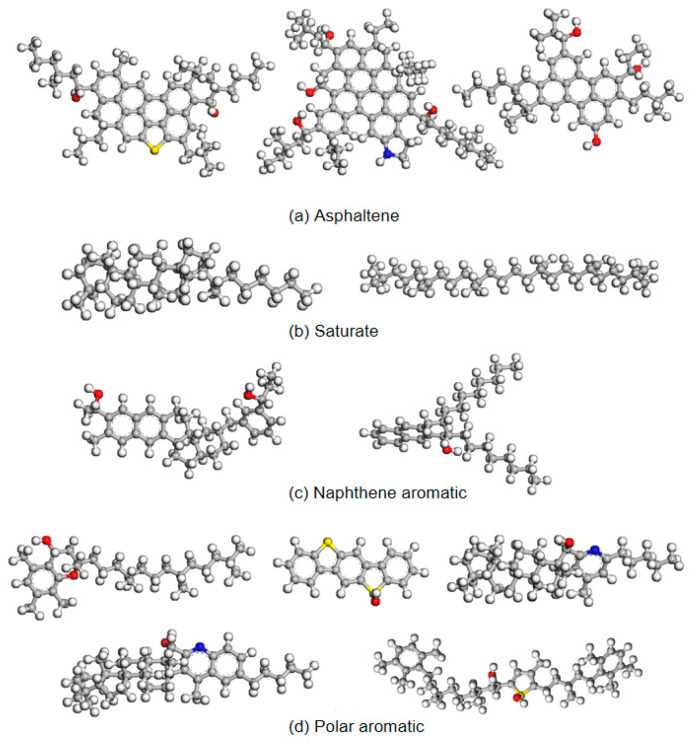
Short-term aged asphalt molecular model (grey for carbon atoms, white for hydrogen atoms, yellow for sulfur atoms, red for oxygen atoms, and blue for nitrogen atoms).

**Figure 5:**
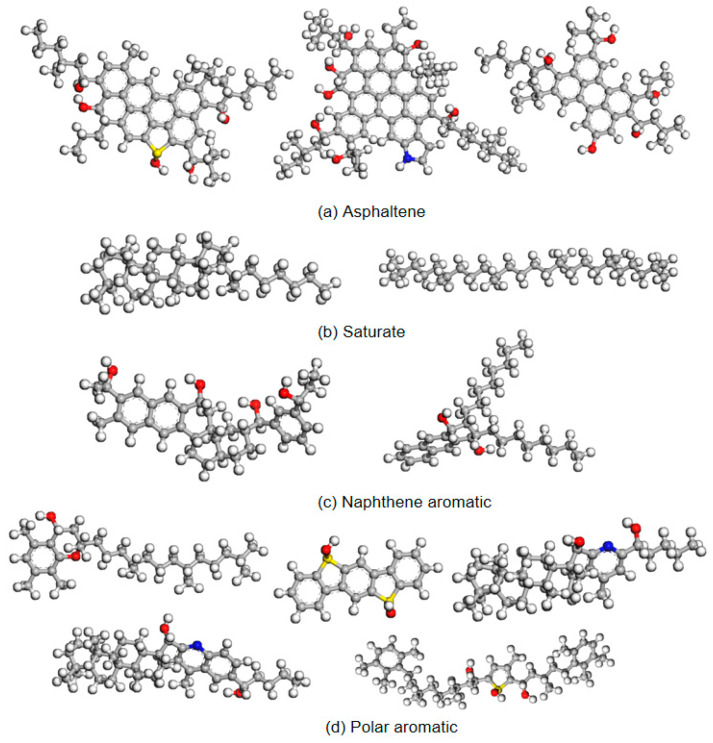
Long-term aged asphalt molecular model (grey for carbon atoms, white for hydrogen atoms, yellow for sulfur atoms, red for oxygen atoms, and blue for nitrogen atoms).

During the processes of mixing, storage, transportation, and paving, asphalt undergoes aging due to exposure to factors such as light, oxygen, and temperature, resulting in a series of chemical and physical changes. Aging of asphalt leads to an increase in its softening point, a decrease in ductility, and significant deterioration in low-temperature performance and fatigue resistance. Throughout the aging process, the aromatic components in asphalt transform into resins, which further transform into asphaltenes, while the content of saturated fractions remains largely unchanged. Due to the influence of oxygen, the chemical composition of asphalt undergoes oxidation and dehydrogenation. Ketone groups (C=O) and sulfoxide groups (S=O) are the primary functional groups formed as a result of asphalt oxidative aging. In the oxidation aging process, oxygen atoms in these groups replace hydrogen atoms on the phenyl carbon, as illustrated in Figure 3. In molecular dynamics simulations, by adding different numbers of ketone and sulfoxide functional groups to the asphalt molecules, short-term aged asphalt molecules and long-term aged asphalt molecules can be obtained, as shown in Figure 4 and Figure 5.

### 2.3. Establishment of Graphene-Modified Asphalt Model

**Figure 6:**
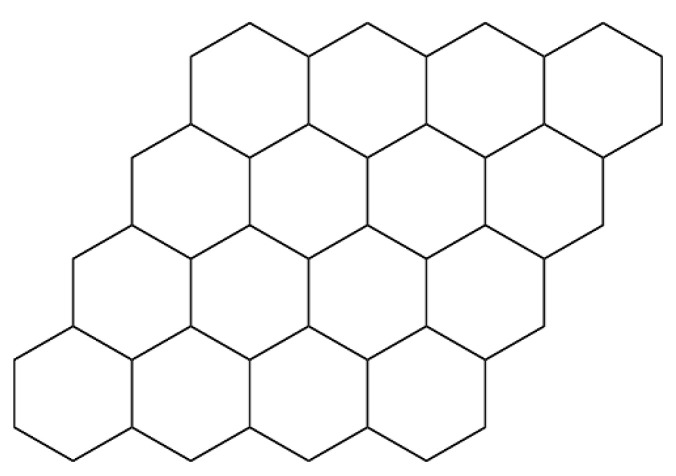
Graphene molecule.

From a microscopic perspective, graphene is a simple yet unique two-dimensional material, consisting of carbon atoms bonded in a single-layer hexagonal honeycomb lattice through sp^2^ hybridization, as shown in Figure 6. By incorporating graphene molecules into the base asphalt model and performing processes such as geometric optimization and dynamic simulation, a graphene-modified asphalt molecular model is obtained.

### 2.4. Validation of the Asphalt Molecular Model

#### 2.4.1. Density

Density is a crucial thermodynamic parameter used to verify whether the chosen force field and model size can meet simulation requirements and produce acceptable and accurate simulation results. By setting both the system simulation temperature and the actual experimental temperature to 25 °C, the reasonableness of the established asphalt molecular model can be judged by comparing the density differences between the two, as shown in Table 2. In the table, 70# refers to base asphalt, GMA refers to graphene-modified asphalt, RTFOT refers to short-term aging, and PAV refers to long-term aging.

From the data in the table, it can be observed that the measured densities are consistently higher than the simulated densities. This discrepancy arises because the asphalt molecular model is constructed under ideal conditions. Some impurities present in real asphalt are not represented in the model, leading to lower simulated densities compared to actual densities. Despite this, the difference between the simulated and real densities does not exceed 6%, which is within the expected range. This demonstrates that the structure of the asphalt molecular model is reasonable and can be used to represent real asphalt.

#### 2.4.2. Cohesion Energy Density

Cohesive energy density (CED) is a physical quantity used to evaluate the intermolecular forces, reflecting the interactions between functional groups. The greater the polarity of the functional groups within the molecule, the stronger the intermolecular forces and, consequently, the higher the cohesive energy density. Using the Forcite module in Materials Studio, the cohesive energy density of the asphalt molecular model can be accurately calculated. The calculated cohesive energy density for the asphalt model is 3.617 × 10^8^ J/m^3^, while the experimentally measured results range from 1.689 × 10^8^ J/m^3^ to 5.063 × 10^8^ J/m^3^. The simulation results align well with the experimental data, indicating that the functional groups within the asphalt have strong interactions, resulting in good cohesiveness.

#### 2.4.3. Radial Distribution Function

**Figure 7:**
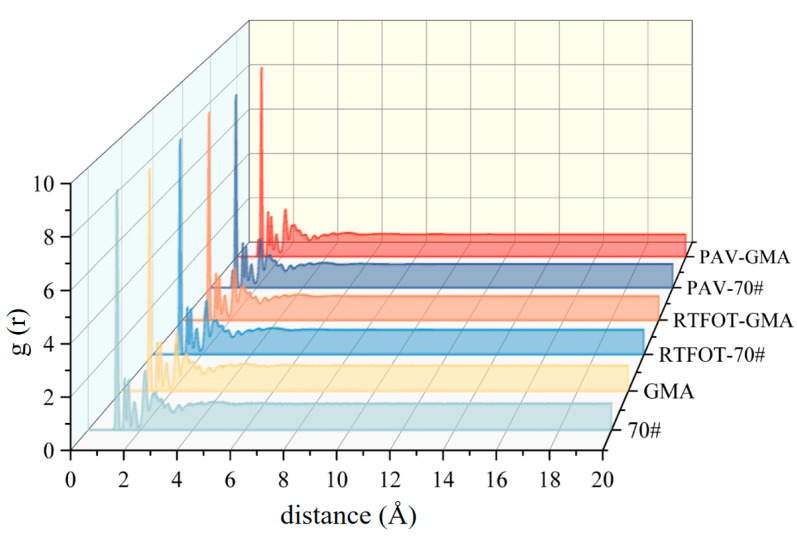
Radial distribution function of the asphalt molecular model.

The radial distribution function (RDF) describes the variation in particle density within a system at a certain distance from a reference particle. Numerically, it is the ratio of the number density of particles within a shell between a maximum radius and a minimum radius to the number density of those particles throughout the entire system. The RDF for the base asphalt molecular model is shown in Figure 7.

In Figure 7, the horizontal axis represents the distance from the reference particle. It can be clearly seen that at distances of 1 Å to 4 Å, the peaks are relatively crowded. As the distance increases to 5 Å, the peaks gradually smooth out and the values approach 1. This indicates that beyond a distance of 5 Å, the particles in the asphalt molecular model are arranged in a disordered manner, suggesting that the asphalt molecular model is an amorphous structure.

#### 2.4.4. Glass Transition Temperature

The glass transition temperature (*Tg*) is an important indicator for assessing the temperature adaptability of asphalt. The transition process of asphalt from a viscoelastic state to a glassy state is known as the glass transition, and the temperature at this transition point is referred to as the glass transition temperature. The *Tg* of the asphalt molecular model can be calculated using molecular dynamics simulations.

**Figure 8:**
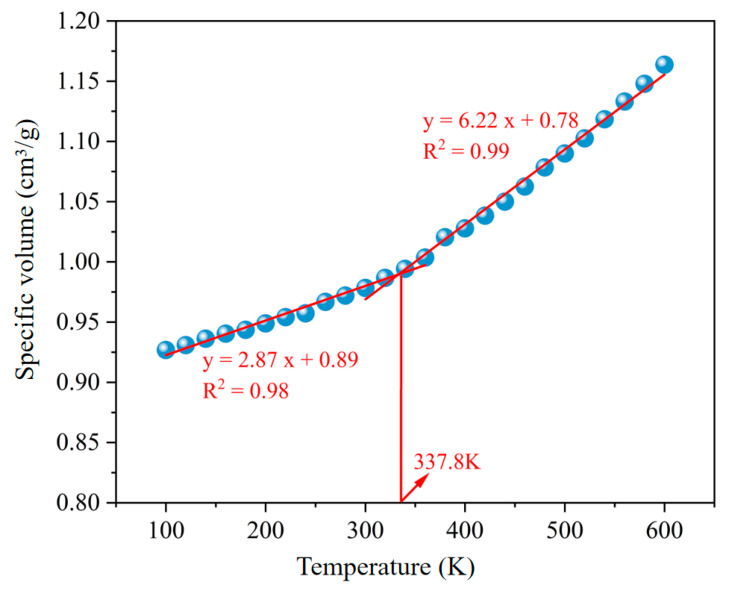
The relation of specific volume of asphalt model with temperature.

Molecular dynamics simulations are conducted at 600 K in the NPT ensemble for 5 ps. After this, a quenching simulation is performed at a cooling rate of 10 K/ns until the temperature is lowered to 100 K. The specific volume of the molecular models obtained through these operations is plotted, and two lines with different slopes are fitted to the data. The *Tg* is located at the intersection of these two lines, as shown in Figure 8. However, due to computational limitations, the *Tg* obtained using this method is often higher than the actual *Tg*. This discrepancy occurs because the cooling rate in the simulation is higher than that in experimental methods, leading to a higher calculated *Tg*. To correct the error caused by the different cooling rates, the Williams–Landel–Ferry (WLF) equation can be used to convert the simulated temperature to the actual temperature. The formula is as Equation (1):

(1) $$\log_{10}\frac{q_{\exp}}{q_{sim}}=\frac{-C_{1}(T_{g,sim}-T_{g,\exp})}{C_{2}+T_{g,sim}-T_{g,\exp}}$$

where *q_exp_* and *q_sim_* are the cooling rates of the experimental and simulation methods, respectively. *C*_1_ and *C*_2_ are constants, with values of 17.44 K and 51.6 K, respectively. *T_g,exp_* and *T_g,sim_* are the glass transition temperatures obtained from the experimental and simulation methods, respectively. Based on Equation (1), the conversion value between the simulation method and the experimental method is calculated to be 99.3 K.

Based on the molecular dynamics simulation method, the *Tg* of the asphalt model is 337.8 K. After subtracting the conversion value of 99.3 K between the simulation and experimental methods, the corrected glass transition temperature is 238.5 K. The experimentally measured glass transition temperature is 245.5 K. The difference between the simulated and experimentally measured *Tg* is within 10 K, indicating that the model is reasonable.

## 3. Construction of Asphalt Crack Model

**Figure 9:**
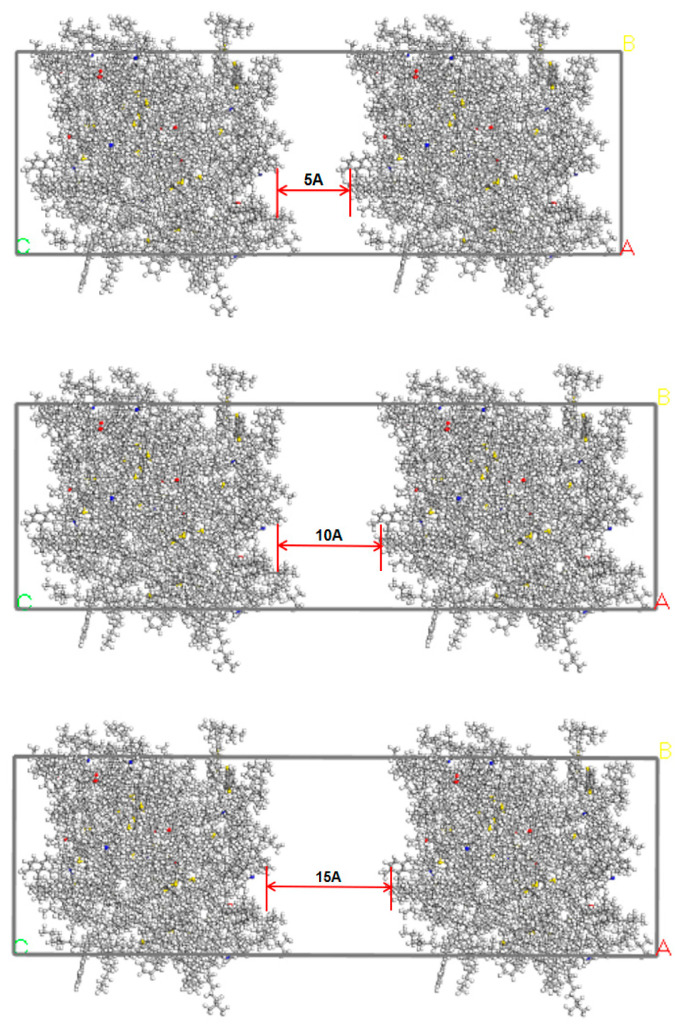
Base asphalt crack mode.

Microcracks are the locations where the self-healing behavior of asphalt materials occurs. To study the microscopic healing mechanism of asphalt, it is essential to first establish an interface model of asphalt. Previous researchers studying the self-healing behavior of asphalt using molecular dynamics mostly chose to artificially insert a specific width of vacuum region between two periodic asphalt models to simulate nanoscale cracks in the asphalt binder [34]. Using the Build Layers tool in Materials Studio 7.0, developed and provided by BIOVIA (San Diego, CA, USA), a three-layer structure of the asphalt crack model is constructed. For studying the healing performance of unaged asphalt, short-term aged asphalt, and long-term aged asphalt, a fixed thickness vacuum layer is introduced between two cells, as shown in Figure 9. After establishing the model, to simulate the self-healing process of asphalt materials, the asphalt–vacuum–asphalt interface model was first structurally optimized for 10,000 steps. Then, using the “Forcite” module, a 200 ps dynamic simulation was performed on the graphene-modified asphalt self-healing model in an NPT system at 1 standard atmosphere. In this study, we used Materials Studio 2019 software for molecular dynamics simulations. The COMPASSⅡ (Condensed-phase Optimized Molecular Potential for Atomistic Simulation Studies) force field was used in this simulation. COMPASSⅡ is an extension and optimization of the COMPASS force field, suitable for a wide range of covalent molecules, including common organic compounds, small inorganic molecules, and polymers. It provides a basis for accurately predicting the material properties of various chemicals. After completing the molecular dynamics simulation, the density changes of the asphalt molecular self-healing model are plotted. Additionally, the “Forcite” module is used to calculate and analyze the mean square displacement (MSD) and diffusion coefficient of the asphalt crack model.

As the self-healing behavior progresses, the density of the asphalt–vacuum–asphalt interface model will gradually increase, approaching the density of undamaged asphalt binder. To clearly observe the diffusion patterns of asphalt molecules around the crack, this study uses asphalt crack widths of 5 Å, 10 Å, and 15 Å as examples to plot the density changes of the asphalt molecular self-healing model under different temperature conditions at 1 atm.

## 4. Molecular Dynamics Simulation Results

Currently, there is no unified set of evaluation criteria for the self-healing performance of asphalt molecular models. Different researchers use various indicators to study the self-healing properties of asphalt models. To validate the accuracy of the simulation results from multiple perspectives, this paper evaluates the self-healing performance of asphalt from both qualitative (density) and quantitative (diffusion coefficient) angles.

### 4.1. Density

**Figure 10:**
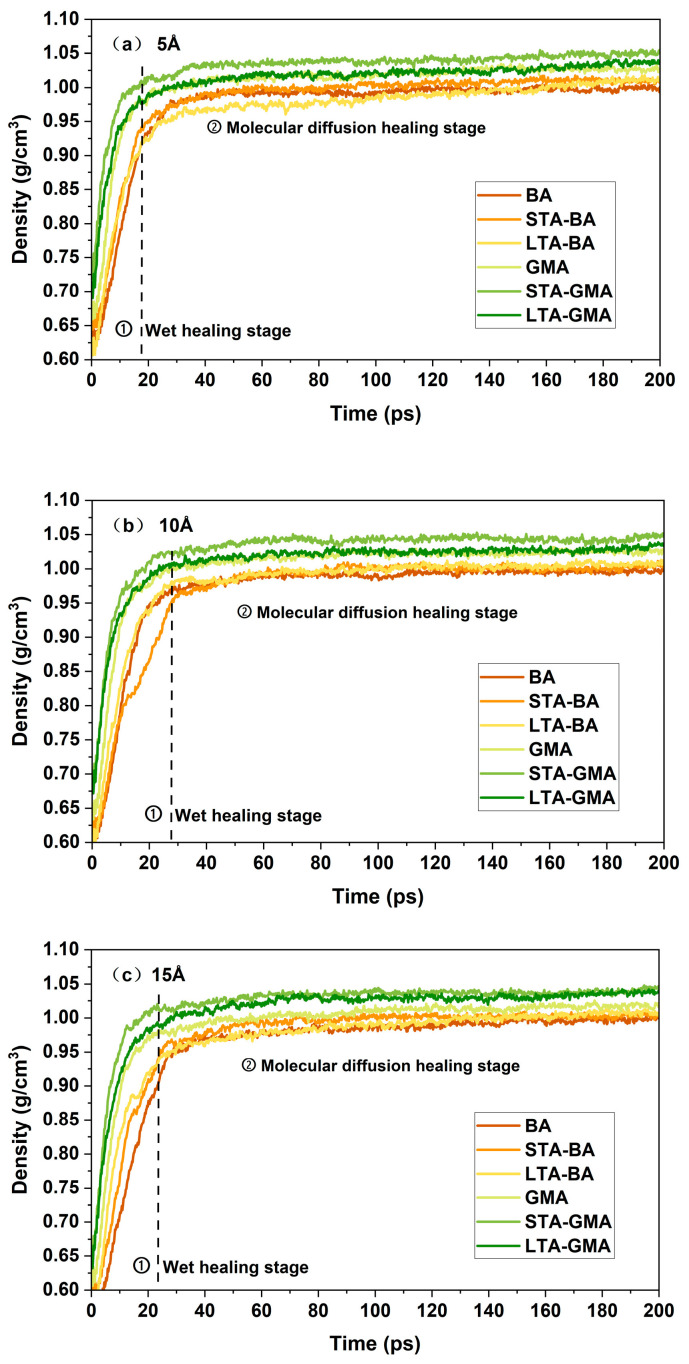
Density of asphalt molecular models with different crack widths: (**a**) 5 Å; (**b**) 10 Å; (**c**) 15 Å.

**Figure 11:**
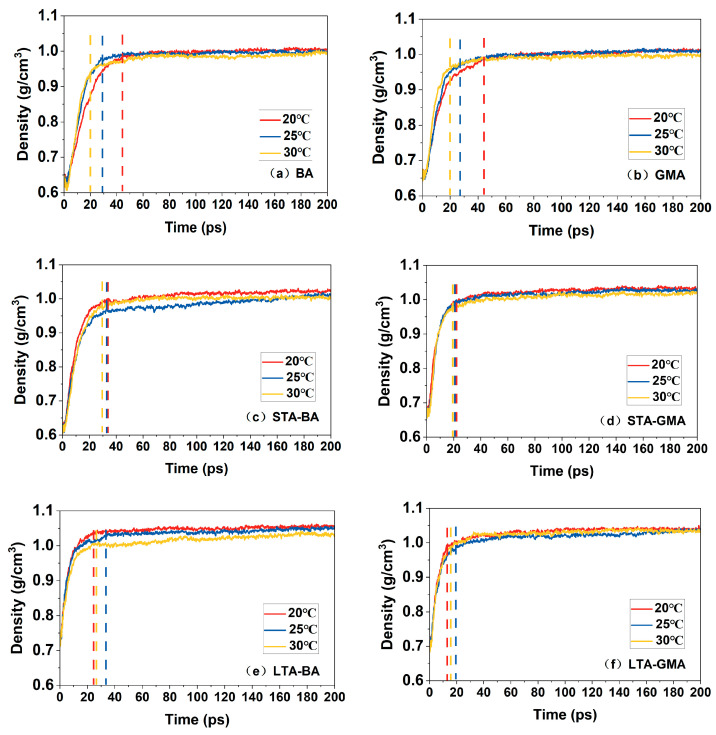
Density of matrix asphalt molecular models at different temperatures (the dashed lines indicate the boundary between the two stages of the asphalt crack model).

Density is a crucial thermodynamic parameter in the molecular dynamics simulation process that reflects the internal physical state of the asphalt crack model. According to polymer self-healing theory, the self-healing process essentially involves the wetting and diffusion of molecules at the crack interface [35]. Although asphalt has a more complex composition, microstructure, and interactions compared to other polymers, its primary self-healing process is very similar to that of polymers [36]. The density of the asphalt model is the most direct fundamental data that reflects the volume changes of the asphalt molecular model over time, directly indicating the healing process of the molecular model. By analyzing the density change curve, the model’s transformation process can be clearly understood. The density variations of asphalt molecular models with different crack widths and temperatures are shown in Figure 10 and Figure 11. In Figure 10 and Figure 11, BA refers to base asphalt, STA-BA refers to short-term aged base asphalt, and LTA-BA refers to long-term aged base asphalt; GMA refers to graphene-modified asphalt, STA-GMA refers to short-term aged graphene-modified asphalt, and LTA-GMA refers to long-term aged graphene-modified asphalt.

Figure 10 illustrates the density changes in asphalt molecular models with different crack widths at 25 °C. It can be observed that the initial density of the asphalt molecular models, due to the introduction of cracks with widths of 5 Å, 10 Å, and 15 Å, ranges between 0.5 g/cm^3^ and 0.7 g/cm^3^. Wider crack widths result in lower initial densities for the asphalt crack models. Additionally, it is evident that the density of the base asphalt is always lower than that of the graphene-modified asphalt, regardless of crack width. To more accurately analyze the healing performance of asphalt, the molecular dynamics healing simulation process can be divided into two stages based on density as an indicator: (1) Wetting Healing Stage: During this stage, the density of the asphalt crack model rapidly increases as the cracks gradually disappear. This process is very similar to the characteristics of the wetting healing stage described in previous studies; (2) Molecular Diffusion Healing Stage: In this stage, the density value stabilizes and becomes very close to that of the undamaged asphalt model. While the density no longer changes significantly, molecular diffusion continues. This stage aligns with the asphalt healing mechanism proposed by Wool and O’Conner, which includes surface rearrangement, surface approach, wetting, diffusion, and randomization [37].

Additionally, it should be noted that during the simulation process, even if the density reaches that of the asphalt molecular model and the visible cracks disappear, the damage within the asphalt is not yet fully healed. The wetting of the asphalt interface can occur almost instantaneously, but the diffusion of asphalt molecules at the crack interface is not yet sufficient. It takes time to reach diffusion equilibrium and randomization, and macroscopically, the strength of the asphalt still requires a longer time to fully recover.

As shown in Figure 11, when the healing temperature varies, the changes in the asphalt crack model can also be divided into two stages: the wetting healing stage and the molecular diffusion healing stage. However, different healing temperatures, degrees of oxidation, and types of asphalt will lead to differences in the duration of these two stages. In Figure 11, the transition points between the two stages for each asphalt crack model are clearly marked. Compared to unaged asphalt, the transition points for short-term aged and long-term aged asphalt at different temperatures do not change significantly, indicating that temperature changes have a greater impact on the healing effect of unaged asphalt.

In the wetting healing stage, increasing the healing temperature leads to an improved density recovery rate and a shorter duration. In the molecular diffusion healing stage, the density stabilizes at a value influenced by the healing temperature. The density inversely correlates with rising temperatures, consistent with general understanding. According to previous research [38], strength recovery primarily occurs in this stage and is highly time-dependent. Regardless of the type of asphalt, as the temperature increases, the duration of the first stage of the asphalt crack model shortens, suggesting that higher environmental temperatures accelerate the healing process of the asphalt crack model to some extent. Under unaged and short-term aged conditions, the transition points for graphene-modified asphalt and base asphalt are very close. However, under long-term aging conditions, the transition points for graphene-modified asphalt occur significantly earlier than those for base asphalt. This indicates that while the healing effects of graphene-modified asphalt and base asphalt are similar under unaged and short-term aged conditions, graphene-modified asphalt performs better under long-term aging conditions. In other words, as the years of use increase, the decline in the healing performance of graphene-modified asphalt is slower than that of base asphalt. Asphalt aging significantly reduces self-healing efficiency, having the greatest impact on healing ability. This reduction is likely due to increased molecular weight and decreased mobility of asphalt molecules. Although healing temperature also affects crack closure rates, its impact on healing efficiency is relatively smaller compared to that of aging. This observation aligns with previous research findings [39].

### 4.2. Mean Square Displacement and Diffusion Coefficient

**Figure 12:**
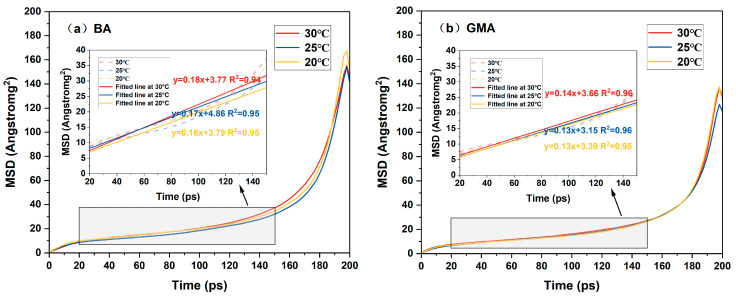
Mean square displacement of asphalt molecular models at different temperatures.

**Figure 13:**
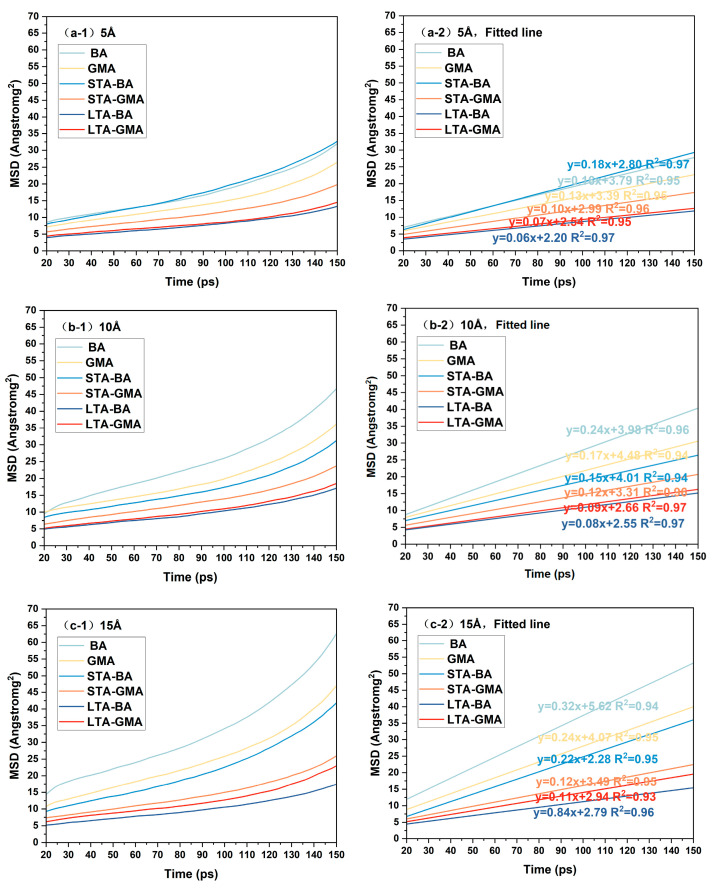
The MSD of asphalt molecular models with different crack widths at 25 °C is analyzed.

Inferring the healing performance of asphalt solely based on the density changes of the asphalt crack model is relatively one-sided. Therefore, this study also calculates the mean square displacement (MSD) and diffusion coefficient of each asphalt crack model, as shown in Figure 12 and Figure 13. In molecular dynamics simulations, for a system in equilibrium, the particles in the system will move according to the set force field and tend to diffuse from their original positions to other locations. The mean square displacement (MSD) of particles can represent the movement of asphalt molecules. The formula for MSD is as Equation (2):

(2) $$m(t)=\sum_{i=1}^{N}\langle {\left|r_{i}(t)-r_{i}(0)\right|}^{2}\rangle$$

where *m*(*t*) is the mean azimuth shift and *r_i_*(*t*) is the position vector of particle *i* at time *t*. The increase in the mean square displacement with time is related to the diffusion coefficient, which can be expressed as Equation (3):

(3) $$D=\frac{1}{6}\underset{t\to \infty }{\lim}\frac{d}{dt}\sum_{i=1}^{N}\langle {\left|r_{i}(t)-r_{i}(0)\right|}^{2}\rangle$$

The limiting slope of the mean square displacement (MSD) can be used to calculate the diffusion coefficient of particles. The diffusion coefficient, calculated from the slope of the MSD curve, has been used to characterize the diffusion or mobility of molecules [40], thereby indicating the self-healing performance of asphalt. The limiting slope refers to the slope of the function that gradually approaches a definite value over a certain interval. This value is typically called the limiting slope. The diffusion coefficient can be simplified as Equation (4):

(4) $$D=\frac{1}{6}a$$

where *a* is the slope of the line fitted by the mean-square displacement function curve.

In Figure 12, it can be observed that the mean square displacement (MSD) of the asphalt crack model is positively correlated with the simulation time, indicating that the movement distance of molecules within the asphalt crack model gradually increases during the molecular dynamics simulation. The slope of the MSD curve can be divided into two stages: a relatively flat stage with a longer duration and a stage where the slope increases sharply. To obtain the value of the diffusion coefficient, this study focuses on the relatively flat first stage data (20 ps to 150 ps). In Figure 12, as the healing temperature increases, the diffusion coefficient of the asphalt crack model also gradually increases. This indicates that, under the same crack width, asphalt type, and aging conditions, the diffusion coefficient steadily rises with higher temperatures, enhancing molecular mobility with more energy provided. This can be explained by the fact that at higher temperatures, more energy is transferred to the asphalt molecular system, allowing molecules to more easily overcome the activation energy barrier and diffuse to the crack interface [37]. Consequently, higher environmental temperatures can improve the healing effect of asphalt.

Comparing the diffusion coefficients of graphene-modified asphalt and base asphalt, it is found that under the same temperature, same crack width, and unaged conditions, the diffusion coefficient of graphene-modified asphalt is lower than that of base asphalt. This means that the movement distance of molecules in the graphene-modified asphalt molecular model is shorter than that in the base asphalt, resulting in poorer healing effects. When the crack width of the asphalt model is small (5 Å), the impact of different temperatures on the diffusion coefficient of the asphalt crack model is minimal, indicating that temperature has little effect on the healing process of asphalt.

Figure 13 shows that when the temperature, crack width, and type of asphalt remain constant, the mean square displacement (MSD) of asphalt decreases with increased aging. This may be related to changes in molecular interactions caused by the increase in polar groups and the reduction in free volume within the asphalt crack model. Consequently, oxidative aging hinders molecular migration within the asphalt crack model, reducing the relaxation ability of the asphalt. As the crack width increases, the diffusion coefficient of the asphalt crack model also increases, indicating that the diffusion behavior of asphalt molecules accelerates. This is because increasing the spacing of the internal vacuum layer in the model during the simulation provides more space for asphalt molecules to diffuse, leading to a higher diffusion rate. Therefore, when studying the variable of crack width, other physical quantities should be considered to avoid the misleading conclusion that wider cracks result in a stronger healing ability of the asphalt model, which contradicts our understanding.

When the crack width of the asphalt model is small (5 Å), the changes in asphalt type and oxidative aging have a minimal impact on the diffusion coefficient of the asphalt crack model, indicating that the disorder-stabilization process of asphalt molecules is less affected. However, as the crack width increases, the impact of asphalt type and oxidative aging on the self-healing process becomes more significant. Specifically, deeper aging or the addition of graphene molecules to the model slows down the self-healing process, increasing the diffusion time of asphalt molecules into the crack.

By combining Figure 12 and Figure 13, it is observed that under different healing temperatures and crack widths, the results of the asphalt crack model are generally consistent. When the crack width is small, factors such as temperature, aging degree, and asphalt type have less impact on the diffusion coefficient results of the asphalt crack model, and the curves are more similar. As the crack width increases, the influence of these factors on the diffusion coefficient of the asphalt crack model becomes more pronounced. In other words, when the crack width increases, the dependence of the asphalt healing effect on various influencing factors is stronger. This may be due to the fact that during the self-healing process, asphalt molecules on both sides of the crack gradually diffuse into the crack and entangle with each other, helping to narrow the crack width. As the crack width increases, the extended travel distance of particles and weakened interactions of asphalt molecules at the crack interface amplify the impact of various factors on the healing effect, making crack closure more difficult. Therefore, crack width, or damage degree, has the greatest influence on the diffusion coefficient of the asphalt crack model.

In summary, the degree of asphalt damage affects its initial density; higher damage levels result in lower initial density of the asphalt crack model, and various factors have a greater impact on the healing effectiveness of the asphalt cracks. Different temperatures have a more pronounced effect on the healing performance of unaged asphalt, with higher temperatures improving healing efficiency. Oxidative aging hinders molecular migration within the asphalt crack model, leading to decreased healing performance, while graphene-modified asphalt demonstrates superior anti-aging properties compared to base asphalt.

## 5. Conclusions

In this study, the Materials Studio software was used to investigate the effects of aging degree, healing temperature, and crack width on the self-healing performance of graphene-modified asphalt. Four parameters—density, radial distribution function analysis, glass transition temperature, and cohesive energy density—were selected to validate the rationality of the established stable asphalt molecular model. The density, mean square displacement, and diffusion coefficient of the asphalt model were used to jointly evaluate the self-healing performance of different asphalt crack models, leading to the following main conclusions:

(1) The healing simulation process can be divided into two stages: the wetting healing stage and the molecular diffusion healing stage. It was found that temperature changes have a greater impact on the healing effect of unaged asphalt. When other conditions remain constant, an increase in healing temperature shortens the duration of the first stage, indicating that the healing process is accelerated to some extent with higher environmental temperatures.
(2) During oxidative aging, the chemical composition of asphalt undergoes oxidation and dehydrogenation, with oxygen atoms in some groups replacing hydrogen atoms on the phenyl carbon. This hinders molecular migration within the asphalt crack model, reducing the relaxation ability of asphalt molecules and ultimately worsening the healing effect. Oxidative aging has a negative impact on self-healing efficiency, showing a higher degree of influence compared to healing temperature.
(3) Under unaged and short-term aging conditions, the healing performance of graphene-modified asphalt is similar to that of base asphalt. However, under long-term aging conditions, the healing performance of graphene-modified asphalt is superior. As the years of use increase, the decline in the healing performance of graphene-modified asphalt is slower than that of base asphalt, indicating stronger anti-aging properties for graphene-modified asphalt.
(4) When the crack width is small, factors such as temperature and aging degree have less impact on the diffusion coefficient results of the asphalt crack model, resulting in similar curves. As the crack width increases, the influence of these factors on the diffusion coefficient becomes more significant. When the crack width is large, the healing effect of asphalt is more dependent on various influencing factors. Increasing crack width amplifies the impact of various factors on the self-healing process, thereby reducing self-healing efficiency.

## Figures and Tables

**Table 1:** AAA-1 asphalt molecular model details.

Molecular Name	Molecular Formula	Atomic Number	Molar Mass (g/mol)	Mass Fraction (%)
Squalane	C_30_H_62_	92	422.8	5.19
Hopane	C_35_H_62_	97	482.9	5.93
PHPN	C_35_H_44_	79	464.7	15.68
DOCHN	C_30_H_46_	76	406.7	16.22
Quinolinohopane	C_40_H_59_N	100	553.9	6.79
Thioisorenieratane	C_40_H_60_S	101	573	7.03
Trimethylbenzeneoxane	C_29_H_50_O	80	414.7	6.36
Pyridinohopane	C_36_H_57_N	94	503.9	6.18
Benzobisbenzothiophene	C_18_H_10_S_2_	30	290.4	13.36
Asphaltene-phenol	C_42_H_54_O	97	574.9	5.29
Asphaltene-pyrrole	C_66_H_81_N	148	888.4	5.45
Asphaltene-thiophene	C_51_H_62_S	114	707.1	6.51

**Table 2:** Table of simulated and measured densities of asphalt at 25 °C (g/cm^3^).

Asphalt Type	Simulated Density	Measured Density
70#	0.995	1.016
GMA	1.007	1.023
RTFOT-70#	1.019	1.074
RTFOT-GMA	1.025	1.076
PAV-70#	1.054	1.082
PAV-GMA	1.04	1.091

## Data Availability

The original contributions presented in the study are included in the article; further inquiries can be directed to the corresponding author.

## References

[1] Men B., Li X., Sha T., Guo F., Tang G., Yue J. (2024). Research on Fatigue Performance of Fast-Melting SBS/Epoxy Resin Composite-Modified Asphalt. Coatings.

[2] Men B., Guo F., Kang X., Yue J. (2023). Research on the Adhesion Properties of Fast-Melting SBS-Modified Asphalt–Aggregate Based on Surface Free Energy Theory. Materials.

[3] Li Y., Wu S., Amirkhanian S. (2018). Investigation of the Graphene Oxide and Asphalt Interaction and Its Effect on Asphalt Pavement Performance. Constr. Build. Mater..

[4] Almutairi H., Baaj H. (2023). Evaluating Self-Healing Behaviour of Asphalt Binders Modified with Phase-Change Materials, Polymers and Recycled Glass Powder. Polymers.

[5] Sun D., Lin T., Zhu X., Tian Y., Liu F. (2016). Indices for Self-Healing Performance Assessments Based on Molecular Dynamics Simulation of Asphalt Binders. Comput. Mater. Sci..

[6] Long Z., You L., Tang X., Ma W., Ding Y., Xu F. (2020). Analysis of interfacial adhesion properties of nano-silica modified asphalt mixtures using molecular dynamics simulation. Constr. Build. Mater..

[7] Hajj R., Bhasin A. (2018). The search for a measure of fatigue cracking in asphalt binders-a review of different approaches. Int. J. Pavement Eng..

[8] Zhu Q., Liu C., Wang Y., Su Y., Li M. (2024). Effect of Modifiers on Self-Healing and Rheological Properties of Asphalt Binder. Materials.

[9] Zhang F., Sun Y., Kong L., Cannone Falchetto A., Yuan D., Wang W. (2024). Study on Multiple Effects of Self-Healing Properties and Thermal Characteristics of Asphalt Pavement. Buildings.

[10] Xu S., Liu X., Tabaković A., Schlangen E. (2021). Experimental Investigation of the Performance of a Hybrid Self-Healing System in Porous Asphalt under Fatigue Loadings. Materials.

[11] Li J., Ji X.-P., Fang X.-Z., Hu Y.-L., Hua W.-L., Zhang Z.-M., Shao D.-Y. (2022). Self-healing performance and prediction model of microcapsule asphalt. Constr. Build. Mater..

[12] de Oliveira L.S., Júnior J.L.O.L., Babadopulos L.F.A.L., Soares J.B. (2022). Stiffness and fatigue evaluation in cyclic tests with rest periods for asphalt mixtures with or without fly ash. Constr. Build. Mater..

[13] Xu S., Tabaković A., Liu X., Palin D., Schlangen E. (2019). Optimization of the Calcium Alginate Capsules for Self-Healing Asphalt. Appl. Sci..

[14] Nalbandian K.M., Carpio M., González A. (2021). Analysis of the scientific evolution of self-healing asphalt pavements: Toward sustainable road materials. J. Clean. Prod..

[15] Li R., Yu S., Chen H., Wu J., Chen Y., Yue J. (2024). Effects of a Complex Environment on Fatigue and Self-Healing Characterization of Asphalt Composites Containing Rock Asphalt. Materials.

[16] Zhang L., Hoff I., Zhang X., Liu J., Yang C., Wang F. (2023). A Methodological Review on Development of Crack Healing Technologies of Asphalt Pavement. Sustainability.

[17] Fan S., Zhu H., Yuan H., Chen C. (2021). Fracture-healing properties of asphalt mixtures and microwave heating thermo-sensitivity analysis of their constituent materials. J. Clean. Prod..

[18] Bhasin A., Ganesan V. (2017). Preliminary investigation of using a multi-component phase field model to evaluate microstructure of asphalt binders. Int. J. Pavement Eng..

[19] Sun D., Sun G., Zhu X., Pang Q., Yu F., Lin T. (2017). Identification of wetting and molecular diffusion stages during self-healing process of asphalt binder viafluorescence microscope. Constr. Build. Mater..

[20] Zhang D.-R., Luo R., Chen Y., Zhang S.-Z., Sheng Y. (2016). Performance Analysis of DCLR-modified Asphalt Based on Surface Free Energy. Zhongguo GongluXuebao/China J. Highw. Transp..

[21] Xiao M., Guo X., Dong J., Li C., Ren J. (2024). Microscopic self-healing of multi-walled carbon nanotube-modified asphalt based on the dual diffusion-energy theory. Can. J. Civ. Eng..

[22] Qu X., Wang D., Wang L., Huang Y., Hou Y., Oeser M. (2018). The state-of-the-art review on molecular dynamics simulation of asphalt binder. Adv. Civ. Eng..

[23] Li C., Wu S., Chen Z., Tao G., Xiao Y. (2018). Improved Microwave Heating and Healing Properties of Bitumen by Using Nanometer Microwave-Absorbers. Constr. Build. Mater..

[24] Liu J., Hao P., Dou Z., Wang J., Ma L. (2021). Rheological, Healing and Microstructural Properties of Unmodified and Crumb Rubber Modified Asphalt Incorporated with Graphene/Carbon Black Composite. Constr. Build. Mater..

[25] Wang R., An X. (2024). An Optimized Fatigue Model of Asphalt Binder Combining Nonlinear Viscoelastic and Intrinsic Healing Characteristics. Constr. Build. Mater..

[26] Wang R., Qi Z., Li R., Yue J. (2020). Investigation of the Effect of Aging on the Thermodynamic Parameters and the Intrinsic Healing Capability of Graphene Oxide Modified Asphalt Binders. Constr. Build. Mater..

[27] Gong Y., Xu J., Yan E.-h., Cai J.-h. (2021). The self-healing performance of carbon-based nanomaterials modified asphalt binders based on molecular dynamics simulations. Front. Mater..

[28] Qu X., Wang D., Hou Y., Liu Q., Oeser M., Wang L. (2019). Investigation on self-healing behavior of asphalt binder using a six-fraction molecular model. J. Mater. Civ. Eng..

[29] Sun W., Wang H. (2020). Self-healing of asphalt binder with cohesive failure: Insights from molecular dynamics simulation. Constr. Build. Mater..

[30] Trong D.N., Long V.C., Ţălu Ş. (2020). The Influence of Shape and Matrix Size on the Mechanical Properties of the 2D Epoxy Thin Film by Monte Carlo Simulation Method. J. Appl. Polym. Sci..

[31] Xu H., Xu W., Zheng X., Cao K. (2022). A Multistage Analysis of Asphalt Binder Nanocrack Generation and Self-Healing Behavior Based on Molecular Dynamics. Polymers.

[32] You Z., Mills-Beale J., Foley J.M., Roy S., Odegard G.M., Dai Q., Goh S.W. (2011). Nanoclay-modified asphalt materials: Preparation and characterization. Constr. Build. Mater..

[33] Li D.D., Greenfield M.L. (2014). Chemical compositions of improved model asphalt systems for molecular simulations. Fuel.

[34] He L., Li G., Lv S., Gao J., Kowalski K.J., Valentin J., Alexiadis A. (2020). Self-healing behavior of asphalt system based on molecular dynamics simulation. Constr. Build. Mater..

[35] Agzenai Y., Pozuelo J., Sanz J., Perez I., Baselga J. (2015). Advanced self-healing asphalt composites in the pavement performance field: Mechanisms at the nano level and new repairing methodologies. Recent Pat. Nanotechnolog..

[36] Ayar P., Moreno-Navarro F., Rubio-Gámez M.C. (2016). The healing capability of asphalt pavements: A state of the art review. J. Clean. Prod..

[37] Sun D., Lin T., Zhu X., Cao L. (2015). Calculation and evaluation of activation energy as a self healing indication of asphalt mastic. Constr. Build. Mater..

[38] Bhasin A., Little D.N., Bommavaram R., Vasconcelos K., Little D.N., Bommavaram R., Vasconcelos K. (2008). A Framework to Quantify the Effect of Healing in Bituminous Materials Using Material Properties. Road Mater. Pavement Des..

[39] Li Y., Zhang H., Wu Z., Sun B. (2023). Influencing Factors and Evaluation of the Self-Healing Behavior of Asphalt Binder Using Molecular Dynamics Simulation Method. Molecules.

[40] Ren S., Liu X., Lin P., Gao Y., Erkens S. (2022). Review on the Diffusive and Interfacial Performance of Bituminous Materials: From a Perspective of Molecular Dynamics Simulation. J. Mol. Liq..

